# Cognitive Reserve in Dementia: Implications for Cognitive Training

**DOI:** 10.3389/fnagi.2016.00084

**Published:** 2016-04-26

**Authors:** Sara Mondini, Ileana Madella, Andrea Zangrossi, Angela Bigolin, Claudia Tomasi, Marta Michieletto, Daniele Villani, Giuseppina Di Giovanni, Daniela Mapelli

**Affiliations:** ^1^Department of General Psychology, University of PaduaPadova, Italy; ^2^Neuromotor Rehabilitation Unit, Casa di Cura Figlie di San CamilloCremona, Italy; ^3^Human Inspired Technology Research Centre, University of PaduaPadova, Italy; ^4^IRCCS Centro S. Giovanni di Dio FatebenefratelliBrescia, Italy

**Keywords:** cognitive reserve, dementia, Alzheimer’s disease, cognitive training, rehabilitation

## Abstract

Cognitive reserve (CR) is a potential mechanism to cope with brain damage. The aim of this study was to evaluate the effect of CR on a cognitive training (CT) in a group of patients with dementia. Eighty six participants with mild to moderate dementia were identified by their level of CR quantified by the CR Index questionnaire (CRIq) and underwent a cycle of CT. A global measure of cognition mini mental state examination (MMSE) was obtained before (T0) and after (T1) the training. Multiple linear regression analyses highlighted CR as a significant factor able to predict changes in cognitive performance after the CT. In particular, patients with lower CR benefited from a CT program more than those with high CR. These data show that CR can modulate the outcome of a CT program and that it should be considered as a predictive factor of neuropsychological rehabilitation training efficacy in people with dementia.

## Introduction

The whole combination of variables including education, intelligence, cognitive learning and knowledge that one acquires throughout life is known as cognitive reserve (CR). The wider the spectrum of experiences and learning, the better the coping with brain damage (Stern, 2009, 2012). CR seems to have a relevant role in the context of degenerative diseases and it may explain the discrepancy between clinical manifestations and the underlying cerebral pathology.

Katzman et al. (1989) first reported findings on interesting incongruities between cognitive symptoms and the underlying neurological disease. Post-mortem examinations revealed that the brains of ten women showed clear histopathological signs of dementia, despite the absence of clinical cognitive signs in life. The authors attributed this incongruity to the bigger size of their brains—reflecting a greater number of neurons—later defined as brain reserve (BR; Stern, 2009, 2012). Mortimer et al. (2003) estimated that between 43% and 67% of individuals reach the threshold for a positive diagnosis of dementia at autopsy despite showing no signs of cognitive decline in life. BR is therefore a passive quantitative model of reserve. In addition, recent neuroimaging studies highlighted that more indirect indexes of neuropathology, such as anatomical changes (Liu et al., 2012), cerebral blood flow reduction (Kemppainen et al., 2008) and metabolic alterations (Hanyu et al., 2008) are found in people with higher level of CR suffering from dementia, despite their quite normal cognitive performance.

CR is an active mechanism which facilitates the flexible use of available BR through efficient information processing and strategies (Barulli and Stern, 2013; Xu et al., 2015). People with higher CR can cope with neuronal damage better than people with lower CR matched for level of pathology and BR (Steffener and Stern, 2012). CR may thus facilitate cognitive performance even in brain impairment and act as a dynamic, flexible and “plastic” mechanism in the brain.

This interpretation is consistent with data on the effects of an enriched environment in animal models in promoting neurogenesis and releasing brain-derived neurotrophic factor (BDNF), which is fundamental for plasticity and brain changes (Brown et al., 2003; Nithianantharajah et al., 2009). Neuroplasticity is the ability of the brain to change according to environmental stimulations or after experiencing neurological damage (Wolf et al., 2006; Bosch et al., 2010). Indeed, living in an enriched environment seems to offset the cognitive decline due to Alzheimer’s disease (AD; Tucker and Stern, 2011).

CR is a theoretical construct that can be quantified through indirect proxies, such as education, occupational attainment and leisure time activities. Data from the literature provide evidence of a reduced risk of AD in people with high educational level (Scarmeas et al., 2004; Allegri et al., 2010). Some protective effects are found for occupations implying high responsibility (Qiu et al., 2003; Wajman and Bertolucci, 2010) and for leisure time activities involving learning, attention, memory and other intellectual skills (Fabrigoule et al., 1995; Wilson et al., 2013). Nevertheless, there is evidence that individuals with high CR have lower life expectancy and quicker progression of the disease after the onset of clinical symptoms of neurodegenerative disease (Hall et al., 2007). At first sight this appears to be counterintuitive, but it is consistent with neuroimaging studies showing a more extended cerebral pathology for high CR than low CR people with similar clinical severity (Hua et al., 2008; Vemuri et al., 2011). Thus, it is reasonable to suppose that, compared with low CR patients with AD, high CR patients’ brain capacity to re-organize networks and learn new information is reduced due to a more extended pathology.

This hypothesis has clear implications in the context of cognitive rehabilitation. Nowadays, findings on cognitive interventions in dementia are of great interest because of the documented existence of a certain degree of neuroplasticity even in the elderly and because of poor evidence of pharmacological treatment efficacy (Cotelli et al., 2012). Researchers are thus encouraged to focus their studies on non-pharmacological interventions. In the last decade many protocols of cognitive training (CT) intervention for AD patients have been validated and their efficacy proved (Yu et al., 2009; Bergamaschi et al., 2013; Mapelli et al., 2013). A set of tasks designed to involve cognitive functions, such as attention, memory, language and problem solving is typically included in any CT and combined with a reality orientation session. The training can be administered either individually or in group sessions with increased levels of difficulty. The belief is that practice with specific cognitive function tasks may improve, or at least maintain, functioning in a given domain and that any effects of practice can be generalized and can produce some improvement of cognitive and social functioning.

Not all the patients involved in these studies obtained the same benefits, probably because of the great inter-individual variability in the elderly (Gorus et al., 2008). To explain this variability many factors have been proposed, such as insight level, severity of behavioral disturbances and comorbidity with other pathologies (Zanetti et al., 2002; Binetti et al., 2013).

Other authors have signaled CR as a factor to take into account when evaluating the efficacy of CT (Olazarán et al., 2004; Berlucchi, 2011; Dorbath et al., 2013; Robertson, 2013). Olazarán et al. (2004) described an interesting effect of CR in old people with cognitive decline. They tried to validate the efficacy of a cognitive-motor intervention in people with minor cognitive impairment (MCI) and early AD and found that less educated benefited more from the rehabilitation program than the more educated. The authors explained their findings by referring to the CR paradigm: given a similar level of clinical symptoms, AD could be more advanced in more educated people and thus could limit their learning ability.

The present work endeavors to investigate the influence that CR might have in modulating the efficacy of a rehabilitation program in AD patients. Assuming that in high CR patients the underlying pathology could be more advanced, the learning process could be prevented or weakened. We hypothesized a cognitive treatment to have more positive outcomes in lower CR patients than in higher CR patients.

## Materials and Methods

### Participants and Procedure

A total of 86 participants aged 61–91 years, all community-dwelling people with mild to moderate dementia, were recruited from four different geriatric clinics in Northern Italy. Participants underwent a deep neurological examination, which included either magnetic resonance or computed tomography scans and a complete neuropsychological evaluation. All of them met the diagnostic criteria of the 5th revision of the diagnostic and statistical manual (DSM-5) for probable major NCD due to AD (American Psychiatric Association, 2013) and the NINCDS-ADRDA criteria for probable AD (Dubois et al., 2007). Of all our subjects, 63 of them received a diagnosis of probable AD and the other 23 were diagnosed with mixed dementia. All patients were able to withstand a formal evaluation, and did not have behavioral disorders that could compromise the CT intervention. Participants and/or caregivers gave their written informed consent for participation in the study, which was approved by the Ethical Committee for the Psychological Research of the University of Padua. In order to evaluate the efficacy of CT we used a global measure of cognition, the Mini—mental state examination (MMSE; Folstein et al., 1975). The use of this single quantitative score was done on purpose because it met the object of the study, having a score on the global cognitive status.

Participants’ level of CR was quantified by the CR Index questionnaire (CRIq; Nucci et al., 2012). The CRIq was administered to their caregivers (a family member), considering that memory and awareness deficits are typical of dementia. Table 1 shows demographic characteristics of all participants and Table 2 reports their clinical data.

**Table 1:** **Demographic characteristics of 86 participants (75% female) at baseline**.

	Mean (SD)
Age	77.98 (7.42)
Education	6.56 (3.31)

*SD, standard deviation*.

**Table 2:** **Psychometric evaluation of patients at baseline**.

Test	Mean (SD)
MMSE	20.26 (2.79)
CRI-Total	94.62 (18.0)
CRI-Education	94.7 (11.95)
CRI-WorkingA.	91.22 (18.62)
CRI-LeisureT.	102.09 (23.47)

*MMSE, mini mental state examination; CRI-Total, cognitive reserve index otal score; CRI-Education, cognitive reserve index education score; CRI-WorkingA., cognitive reserve index working activities score; CRI-LeisureT., cognitive reserve index leisure time activities score*.

The MMSE was administered to all participants before the treatment (T0) to assess their level of cognitive impairment, and after the treatment (T1) in order to evaluate the change in their performance after a cycle of CT. Participants underwent a thorough neuropsychological assessment, but for the reasons explained above we will here report only the MMSE score, which is the most widely used and reliable instrument to assess global cognition in dementia. All patients had at baseline a cognitive status considered as mild to moderate dementia (MMSE; Mean = 20, 26; SD = 2, 79; range 14–25).

It is reported (Tombaugh, 2005) that the mean difference in MMSE test-retest (i.e., the practice effect) in an average time interval of 3 months is negligible in healthy subjects. Thus we administered the same version of the test both before and after the training without expecting any practice effects in patients. Furthermore, as reported by Rush (2000), the test-retest reliability of the MMSE in patients with dementia (most of them with Alzheimer’s type) ranged from 0.75 to 0.94 (Pearson’s *r*) in a number of studies that employed test-retest intervals from 1 day to 9 weeks.

### Cognitive Reserve Index Questionnaire (CRIq)

CR is a complex construct that can be measured by an array of indirect indexes. The literature reports three main proxies of CR: education, occupational attainment, and leisure time activities. However, there is lack of agreement about the importance given to these variables and their quantification. With few exceptions (e.g., Pillai et al., 2012), most studies consider education as the fundamental proxy of CR and most studies converge to this proxy, excluding others, as shown in Meng and D’Arcy’s (2012) systematic meta-analysis.

Nucci et al. (2012) proposed a new tool to measure CR: the CRIq (free download at www.cri.psy.unipd.it). This instrument is a semi-structured interview that gathers and quantifies all the experiences that a person has acquired throughout their life. The CRIq includes demographic data and 20 items grouped into three sections: cognitive reserve index education score (CRI-Education), CRI-Working Activities and CRI-Leisure Time Activities. CRI-Education refers to a person’s school years plus any other educational course (e.g., learning to play a musical instrument or to speak a foreign language). The second section (CRI-Working Activities) considers five categories of working activities with different degrees of responsibility and demands. The number of years spent doing each job is calculated and considered. In the section on “Leisure Time Activities”, information regarding activities done weekly, monthly and yearly are recorded. Low correlation has been reported between these three areas (Nucci et al., 2012), so the information collected by the three sections of the questionnaire does not seem to be redundant and the three proxies can be considered independent of one another. The CR Index (CRI) is the total score resulted from the combination of the sub-scores of each single section, and it is adjusted for age to allow comparisons between groups of different age. The questionnaire requires about 10–15 min to be administered and since it is not a performance test, information can be provided by the patient’s caregiver thus avoiding vagueness or lack of information due to memory loss and poor insight. The questionnaire is a very useful and complete tool to quantify CR in AD patients.

### Cognitive Training

All patients participated in a cycle of CT for a period of 2–4 months. They attended 1 or 2 h sessions once or twice a week, with an expert neuropsychologist, in small groups (5–6 patients in each group). As the CT was administered in different centers and at different times, the length of intervention varied: some patients were trained for 4 months (weekly 1 h sessions), others were trained for 2 months (weekly 2 h sessions). However, all patients received the same amount of training. The types of activities carried out were very similar to the CT described in the literature which has been reported in detail elsewhere (Bergamaschi et al., 2013; Mapelli et al., 2013). All paper-and-pencil exercises were taken from Bergamaschi et al. (2008) and Iannizzi et al. (2015). The specific exercises were designed to stimulate spatial and temporal orientation, memory, attention, language, perception, and visual analysis. This type of CT is the same described in Mapelli et al. (2013) and in Bergamaschi et al. (2013), and has proven its efficacy in decreasing severity of dementia and behavioral symptoms as well as in improving cognitive performance.

Each CT session started with personal, spatial and orientation tasks and continued with some exercises for the structured stimulation of cognitive domains. The specific exercises were designed to stimulate cognition and recognize emotional expressions. For example, in the time orientation exercises the patients had to perform recognition and naming tasks of pictures of clothing and tasks in which they had to associate clothing with pictures of the four seasons. In spatial orientation tasks the patients had to imagine going into different rooms of a house drawn on a plan and indicate the direction (left, right, etc.) of their movements. In the logical reasoning exercises the patients were asked either to complete simple puzzles or rearrange sentences in a logical sequence (e.g., I went to the supermarket, I bought vegetables, I cooked a soup, I ate it, I washed the dishes). Throughout the training, the difficulty of the CT exercises was gradually increased.

The aim of the present study is to ascertain the possible influence of CR on the outcomes of a cognitive rehabilitation program for patients with dementia.

### Statistical Analysis

The considered dependent measure was the difference in MMSE scores between T1 and T0.

Thus three types of results were possible: (a) a positive value indicating improvement; (b) a negative value indicating worsening; and (c) a value equal to zero indicating unchanged performance. Fifty-seven participants (66.3%) improved the MMSE score between T0 and T1 (Mean = 2.67; SD = 1.44), fourteen (16.3%) worsened (Mean = −1.36; SD = 0.49) and fifteen (17.4%) showed no differences. The differences ranged from −2 to +7. High differences in MMSE score (ranging from −4 to +10) in a 3 months’ interval test-retest have been assessed on healthy individuals, as well (e.g., Tombaugh, 2005). We then performed multiple regression analyses to investigate the relation between the dependent variable, age and CR measures. The years of education were not considered since this information was taken into account by the CRI-Education. A total of seventeen models were built. The null model—Model 0—contained the intercept only. From Model 1 to 3, age and cognitive reserve index total score (CRI-Total) were considered as predictors: Model 1 included age only, Model 2 included CRI-Total score only and Model 3 included both age and CRI-Total score.

Then, from Model 4 to 17 the CRI sub-section scores were considered (CRI-Education, CRI-Working Activity and CRI-Leisure Time) and all the combinations of age and CRI sub-sections as predictors were evaluated (Table 3).

**Table 3:** **Linear regression models compared through BIC, AIC, BF and *R*^2^**.

	Dependent variable: difference T1–T0 in MMSE score					
	**Model**	**BIC**	**AIC**	**BF**	***R*^2^**	Model *p*
	Model 0 intercept	372.52	367.61	–	–	n.s.
**CRI-Total score**	Model 1 age	376.97	369.61	0.23	–	n.s.
	**Model 2 CRI-Total**	**362.27**	**354.9**	**148.73**	**0.16**	**< 0.001**
	Model 3 age + CRI-Total	366.71	356.89	36.43	0.16	< 0.001
**CRI sub-sections score**	Model 4 CRI-Ed	372.76	365.40	1.39	0.05	0.043
	Model 5 age + CRI-Ed	376.87	367.05	0.48	–	n.s.
	Model 6 CRI-WA	367.92	360.55	11.79	0.10	0.003
	Model 7 age + CRI-WA	372.37	362.55	3.21	0.10	0.013
	Model 8 CRI-Ed + CRI-WA	372.00	362.19	3.75	0.10	0.011
	Model 9 age + CRI-Ed + CRI-WA	376.43	364.16	1.27	0.10	0.029
	Model 10 CRI-LT	367.96	360.60	11.57	0.10	0.003
	Model 11 age + CRI-LT	372.11	362.29	3.59	0.10	0.011
	Model 12 CRI-Ed + CRI-LT	370.84	361.03	6.15	0.12	0.006
	Model 13 age + CRI-Ed + CRI-LT	375.28	363.01	2.03	0.12	0.017
	**Model 14 CRI-WA + CRI-LT**	**366.41**	**356.59**	**41.49**	**0.16**	**< 0.001**
	Model 15 age + CRI-WA + CRI-LT	370.61	358.33	13.98	0.16	0.002
	Model 16 CRI-Ed + CRI-WA + CRI-LT	370.85	358.58	12.61	0.16	0.002
	Model 17 age + CRI-Ed + CRI-WA + CRI-LT	375.05	360.32	4.91	0.16	0.006

*CRI-Total, Cognitive Reserve Index Total score; CRI-Ed, Cognitive Reserve Index Education score; CRI-WA, Cognitive Reserve Index Working Activity score; CRI-LT, Cognitive Reserve Index Leisure Time activities score; BIC, Bayesian Information Criterion; AIC, Akaike Information Criterion; BF, Bayes Factor*.

Models considering CRI-Total score (Models 1–3) were compared via a set of indexes providing both absolute and relative goodness-of-fit measures: *R*^2^, Akaike Information Criterion (AIC; Burnham and Anderson, 2002), Bayesian Information Criterion (BIC; Schwarz, 1978) and Bayes Factor (BF; Kass and Raftery, 1995; Lavine and Schervish, 1999). The same procedure was followed comparing models considering CRI sub-section scores (Models 4–17). According with these indexes, the lower BIC and AIC and the higher BF and *R*^2^, the better model fit of the data. All statistical analyses were performed using R Software (R Core Team, 2013)^1^.

## Results

All the previously described models, tested through regression analyses, were significant (*p* < 0.05) apart from Model 1 (with age as predictor) and Model 5 (with age and CRI-Education).

Based on the analysis of AIC, BIC, BF and *R*^2^, the best models (considering CRI-Total score and CRI sub-sections respectively) were identified as those that minimized AIC and BIC and maximized BF and *R*^2^ (Table 3).

**Figure 1:**
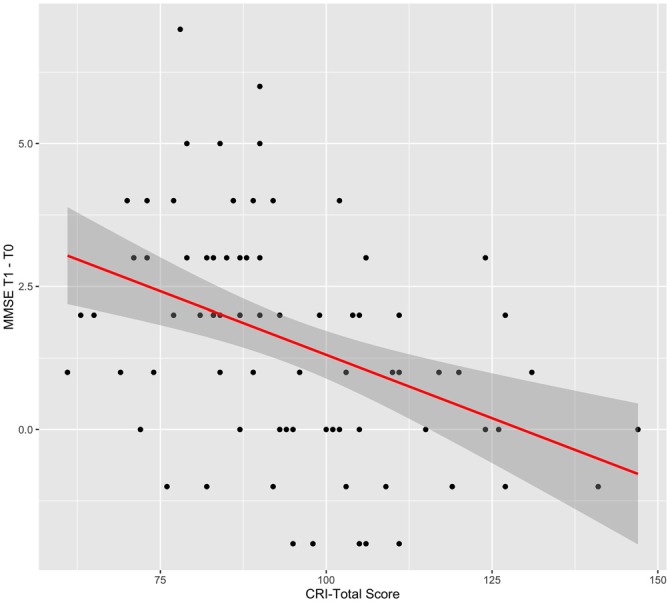
**Effect of cognitive reserve index total (CRI-Total) score.** Graphical representation of the effect of CRI-Total score on the difference in Mini—mental state examination (MMSE) score between T1 and T0, according to Model 2. The line indicates the regression line and the gray area indicates its 95% C.I.

For the CRI-Total score, the best model was Model 2 with CRI-Total score only as predictor (BIC = 362.27; AIC = 354.9; BF = 148.73; *R*^2^ = 0.16; *p* < 0.001). CRI-Total score was a significant influencing factor (*t* = −3.958, *p* < 0.001), with lower CRI-Total participants obtaining higher differences in MMSE scores between T1 and T0, if compared with higher CRI-Total participants (Figure 1).

**Figure 2:**
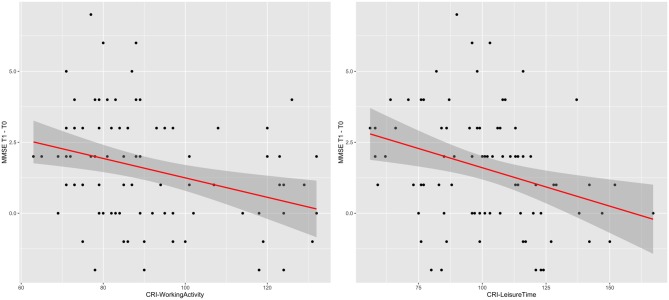
**CRI-Working Activity and CRI-Leisure Time effects.** Graphical representation of the effects of CRI-Working Activity and CRI-Leisure Time on the difference in MMSE score between T1 and T0, according to Model 14. The line indicates the regression line and the gray area indicates its 95% C.I.

For CRI sub-sections, Model 14 was the best model (BIC = 366.41; AIC = 356.59; BF = 41.49; *R*^2^ = 0.16; *p* < 0.001) with CRI-Working Activity (*t* = −0.028; *p* = 0.017) and CRI-Leisure Time (*t* = −0.022; *p* = 0.016) as significant predictors. This result indicates that participants with lower CRI-Working Activity and lower CRI-Leisure Time showed higher differences in MMSE scores between T1 and T0, when compared with participants with higher CRI-Working Activity and higher CRI-Leisure Time (Figure 2).

## Discussion

The present study investigated the influence that CR may have in modulating the efficacy of a CT rehabilitation program in people with degenerative disease. Our results show that taking into account age and level of CR, only CR can predict a significant change in patients’ cognitive performance after rehabilitation training. In fact, in patients with similar clinical symptomatology this change takes the form of an improvement only in subjects with lower level of CR, while patients with higher CR do not seem to similarly benefit from the rehabilitation program. We suggest that in high CR patients the underlying pathology may be more advanced, thus their potential learning ability is reduced and diminishes with the progression of the disease.

These findings are consistent with the study by Olazarán et al. (2004) that highlighted an analogous role of CR in the context of rehabilitation. However, while they considered education as the only proxy for the reserve concept, in the present investigation other variables were also taken into account. An important role of working experiences and leisure time activities was found, that is, the other two proxies of CR measured with the CRIq. Our data show that improvement after treatment is inversely related to total CR (the lower CRI-Total index, the better the improvement) and to both occupational attainment (the lower CRI-Working Activities, the better the improvement) and free-time activities (the lower CRI-Leisure Time, the better the improvement). Thus, a CT program seems to be more beneficial for people with lower CR than for people with higher CR.

These results are of great interest because they highlight that CR cannot be equated to education (Meng and D’Arcy, 2012), nor quantified without considering the wider range of experiences and knowledge one acquires over the lifespan (i.e., working activities and leisure time activities). This does not assume that education by itself is irrelevant in the context of CR. Rather, in our study the lack of a significant effect of education (measured with the CRIq, CRI-Education) and cognitive performance after CT could be due to the demographic characteristics of our sample (about 80% attended only 5 years of formal education).

Altogether, our findings show that a CT program for patients with dementia is more beneficial for low CR than high CR patients. This can be explained by a more extended underlying neuropathology in high CR patients which prevents them from learning and improving their performance after a cycle of cognitive stimulation. A high CR initially allows people to cope better with neuropathology and to delay its clinical manifestation, but according to Stern’s model of CR, the development of pathology will eventually lead to the point after which reserve can no longer withstand the pathology. At this inflection point the progression of the disease is dramatically faster in people with high CR than in people with low CR (Stern, 2009).

All these data suggest that the building of reserve takes place along the lifespan, including adulthood, and thus support the presence of brain plasticity across the entire existence (Burgener et al., 2008; Mondini et al., 2014). Furthermore, this highlights that the CR measure must consider all lifespan activities that can stimulate cognition and the amount of CR.

To our knowledge the present work is the first that has investigated the influence of CR in modulating the efficacy of a rehabilitation program in people with degenerative disease. The results are promising. However, notwithstanding the large sample of patients involved in this research, they need to be replicated with the addition of other measures of global cognitive functioning and considering or comparing different types of rehabilitation programs.

## Author Contributions

SM: contribution in collecting data, cohordinating the group and writing the manuscript. IM: contribution in collecting data and writing the manuscript. AZ: contribution in analyzing data and writing the manuscript. AB, CT, MM: contribution in collecting data and writing the manuscript. DV: contribution in selecting patients and writing the manuscript. GDiG: contribution in selecting patients and writing the manuscript. DM, DV: contribution in collecting data and writing the manuscript.

## Funding

This work was supported by University of Padova, Department of General Psychology (ex 60% to SM).

## Conflict of Interest Statement

The authors declare that the research was conducted in the absence of any commercial or financial relationships that could be construed as a potential conflict of interest.

## Footnotes

^1^http://www.R-project.org/
